# Health Workforce Development in Health Emergency and Disaster Risk Management: The Need for Evidence-Based Recommendations

**DOI:** 10.3390/ijerph18073382

**Published:** 2021-03-24

**Authors:** Kevin K. C. Hung, Sonoe Mashino, Emily Y. Y. Chan, Makiko K. MacDermot, Satchit Balsari, Gregory R. Ciottone, Francesco Della Corte, Marcelo F. Dell’Aringa, Shinichi Egawa, Bettina D. Evio, Alexander Hart, Hai Hu, Tadashi Ishii, Luca Ragazzoni, Hiroyuki Sasaki, Joseph H. Walline, Chi S. Wong, Hari K. Bhattarai, Saurabh Dalal, Ryoma Kayano, Jonathan Abrahams, Colin A. Graham

**Affiliations:** 1 Accident and Emergency Medicine Academic Unit, Chinese University of Hong Kong, Hong Kong, China; kevin.hung@cuhk.edu.hk (K.K.C.H.); emily.chan@cuhk.edu.hk (E.Y.Y.C.); makikokato@cuhk.edu.hk (M.K.M.); jwallinemd@gmail.com (J.H.W.); 2 Collaborating Centre for Oxford University and Chinese University of Hong Kong for Disaster and Medical Humanitarian Response (CCOUC), School of Public Health and Primary Care, The Chinese University of Hong Kong, Hong Kong, China; cswong@cuhk.edu.hk; 3 Research Institute of Nursing Care for People and Community, University of Hyogo, Akashi 673-8588, Japan; sonoe_mashino@cnas.u-hyogo.ac.jp; 4 Beth Israel Deaconess Medical Center, Boston, MA 02115, USA; SBalsari@bidmc.harvard.edu (S.B.); gciotton@bidmc.harvard.edu (G.R.C.); ahart1@bidmc.harvard.edu (A.H.); 5 Department of Global Health and Population, Harvard TH Chan School of Public Health, Boston, MA 02115, USA; 6 Harvard Medical School, Boston, MA 02115, USA; 7 CRIMEDIM—Center for Research and Training in Disaster Medicine, Humanitarian Aid and Global Health, Università del Piemonte Orientale, 28100 Novara, Italy; francesco.dellacorte@med.uniupo.it (F.D.C.); marcelo.dellaringa@uniupo.com (M.F.D.); luca.ragazzoni@med.uniupo.it (L.R.); 8 Division of International Cooperation for Disaster Medicine, International Research Institute of Disaster Science (IRIDeS), Tohoku University, Miyagi 980-0845, Japan; egawas@surg.med.tohoku.ac.jp (S.E.); hsasa@surg.med.tohoku.ac.jp (H.S.); 9 College of Nursing, University of the Philippines Manila, Manila 1000, Philippines; bdevio@up.edu.ph; 10 Emergency Medicine, West China Hospital of Sichuan University, Chengdu 610041, China; whowasyoung@hotmail.com; 11 Department of Educational and Support for Regional Medicine, Tohoku University Hospital, Miyagi 980-8574, Japan; t-ishi23@med.tohoku.ac.jp; 12 PhD Scholar at International PhD in Global Health, Humanitarian Aid and Disaster Medicine Jointly Organized by Università del Piemonte Orientale (UPO) and Vrije Universiteit Brussel (VUB), 28100 Novara, Italy; hkrishnabhattarai@gmail.com; 13 World Health Organization Country Office, New Delhi 110011, India; sdalal@who.int; 14 World Health Organization Centre for Health Development, Kobe 651-0073, Japan; kayanor@who.int; 15 Disaster Risk Reduction and Resilience Unit, Health Security Preparedness Department, WHO Health Emergencies Programme, World Health Organization, CH-1211 Geneva, Switzerland; abrahamsj@who.int

**Keywords:** health emergency and disaster risk management (Health EDRM), Health EDRM workforce development, disaster, health emergency

## Abstract

The Sendai Framework for Disaster Risk Reduction 2015–2030 placed human health at the centre of disaster risk reduction, calling for the global community to enhance local and national health emergency and disaster risk management (Health EDRM). The Health EDRM Framework, published in 2019, describes the functions required for comprehensive disaster risk management across prevention, preparedness, readiness, response, and recovery to improve the resilience and health security of communities, countries, and health systems. Evidence-based Health EDRM workforce development is vital. However, there are still significant gaps in the evidence identifying common competencies for training and education programmes, and the clarification of strategies for workforce retention, motivation, deployment, and coordination. Initiated in June 2020, this project includes literature reviews, case studies, and an expert consensus (modified Delphi) study. Literature reviews in English, Japanese, and Chinese aim to identify research gaps and explore core competencies for Health EDRM workforce training. Thirteen Health EDRM related case studies from six WHO regions will illustrate best practices (and pitfalls) and inform the consensus study. Consensus will be sought from global experts in emergency and disaster medicine, nursing, public health and related disciplines. Recommendations for developing effective health workforce strategies for low- and middle-income countries and high-income countries will then be disseminated.

## 1. Introduction

Between 2000 and 2019, 7348 disasters associated with natural hazards were recorded worldwide, resulting in 1.2 million deaths and affecting over 4 billion people [1]. The recent COVID-19 pandemic alone has contributed an additional 2 million deaths by early 2021 [2], with a consistently growing number of infected people due to the COVID-19 virus being highly infectious, including its variants. The pandemic presented an unprecedented challenge to all sectors in countries, including public health, and continues to put significant pressure on health systems and health workforce capacity at local, national, and global levels. The high toll of morbidity and mortality from disasters amplifies the need for countries to increase health system capacity and for the world to develop a coherent view of disaster risk management by adapting to whole-of-government and whole-of-society approaches. 

Disaster risk depends on the complex interaction between the severity and frequency of a hazard, the numbers of people exposed to the hazard, their vulnerability, and risk management capacities. These four factors, (hazard, exposure, vulnerability and capacity) are influenced by various risk determinants, such as poverty, unplanned urbanisation, climate change and state fragility [3], warranting solidarity at national and global levels. In order to reduce global disaster risks, the Sendai Framework on Disaster Risk Reduction 2015–2030 (Sendai Framework) was adopted by the 2015 Third United Nations (UN) World Conference on Disaster Risk Reduction (UNWCDRR), endorsing targets and priorities for disaster risk management (DRM) [4]. Evolving from previous global Disaster Risk Reduction (DRR) frameworks, such as the Yokohama strategy (1994) [5] and the Hyogo Framework (2005) [6], the Sendai Framework places human health at the centre of global DRM and urges all UN member states to take action to strengthen their DRM capacities to protect the lives, livelihoods, and health of their people [7,8]. 

To integrate the concept of one health into DRM, the World Health Organisation (WHO) Thematic Platform for Health Emergency and Disaster Risk Management (Health EDRM) was established in 2009 [9]. The Sendai Framework has catalysed Health EDRM activities: the WHO Thematic Platform for Health EDRM Research Network (Health EDRM RN) was founded in 2016 to promote global research collaboration and to provide technical advice for evidence-based Health EDRM-related policies and practices [10,11,12,13]. These developments reflected the evolution of Health EDRM over the past decade and the publication of the WHO Health EDRM Framework in 2019, involving comprehensive health-related DRM actions across prevention, preparedness, response, and recovery. It also provides policy recommendations on how to reduce health risks, vulnerabilities and exposures and to increase coping capacity for resilience building in health systems, communities, and countries [8,10]. 

The Health EDRM framework is aligned and consistent with the targets and priorities of actions in the Sendai Framework and the 13th WHO General Programme of Work [14] and contributes to other UN landmark agreements, such as the Sustainable Development Goals [15], the Paris Agreement for Climate Change [16], and the International Health Regulations (2005) [17]. These important global frameworks can work in combination to develop more comprehensive actions for protecting human lives, resilience, health security and development [8].

## 2. Health EDRM Workforce

Health EDRM has emerged from a range of disciplines, including risk management, emergency management, epidemic preparedness and response, community disaster resilience and health systems strengthening. Health EDRM is founded on the broad intersection of health and disaster risk management. It facilitates a comprehensive whole-of-society approach to manage risks from all hazards for countries and communities, build stronger capacities and systems across health and other sectors, and reduce the health risks from emergencies and disasters. 

The Health EDRM Framework outlines risk management concepts and 10 components and around 200 functions of effective Health EDRM. One of the core components is human resource management, including planning for staff (e.g., surge capacity for emergency response including rapid response team), training for competency development, and occupational health and safety of personnel including community-level health workforce and as well protection of all. 

The Health EDRM RN also identified health workforce development as one of the key research areas, highlighting the knowledge gaps in a common understanding of relevant knowledge and competencies required for the Health EDRM workforce as well as the contents for both organization and country level training/professional development. This included their interaction with stakeholders, understanding how to sustain the development of the local health workforce for Health EDRM, fostering positive interactions between external support workers and the local workforce and the effective transition to recovery, and integrating measures to reduce risks of future events and build stronger systems. More understanding is needed about how countries can strengthen Health EDRM through disaster risk management training programs, and how they are able to retain, motivate and utilise trained personnel, including for deployment [11]. 

Functioning human resource capacities and management systems are crucial in order to perform these many Health EDRM functions and cope with all possible health risks from all hazards emergencies. To effectively manage and mobilise all available human resources with different levels of skills, experiences, and knowledge, a good understanding of the function and composition of the Health EDRM workforce is necessary. A risk management approach to Health EDRM also recognises that much of the health workforce have roles to play in reducing the risks and impacts of emergencies and disasters. Due to this broad composition of the Health EDRM workforce—the lack of well-established models for administration and governance at the country level and the shared responsibilities for fulfilling functions—there are intrinsic difficulties in using workforce groups as a basis for providing a robust taxonomy for the Health EDRM workforce.

Human resources for health are broadly defined by the WHO (2006) as “all people engaged in actions whose primary intent is to enhance health” [18]. This definition includes all health professionals, such as physicians, nurses, pharmacists, and other professionals (e.g., managers, ambulance personnel and administrative staff) who are also essential for maintaining functional health systems [19]. They are, undoubtedly, vital workforce groups in the Health EDRM context. During hazardous events, additional groups, such as rescue personnel and community workers, also have important roles in saving people’s lives. Hence, they should also be included in Health EDRM workforce development strategies, for example, in surge capacity planning. 

## 3. Research Needs for Health EDRM Workforce Development

In order to develop an effective Health EDRM workforce at local, national and global levels, we must first acknowledge that major gaps exist in workforce development and evidence [11,20].

First, it is important to identify agreeable definitions for the Health EDRM workforce in the context of the different components of health systems. That may help to characterise the profile and scope of those involved (e.g., health service delivery, policy, planning and coordination, human resource management, financing, logistics, community-based Health EDRM). 

Second, various educational and training programmes have been developed worldwide by academic institutions, hospitals, professional bodies, governments, and non-governmental organisations, which address health systems and healthcare professionals’ ability to manage the risks associated with emergencies. However, these programmes tend to use competencies, terminologies and course structures from only a single programme or institution, and some may not have been developed based on research evidence [20,21,22,23]. Hence these programmes may vary widely and the inconsistency between programmes has hampered risk management, including prevention, preparedness, response, readiness, recovery and coordination during emergencies, sometimes resulting in fragmented assistance to affected communities [24,25,26]. 

Furthermore, the need to engage community health workers (CHWs) at all disaster phases became prominent during the 2014–2016 Ebola virus outbreak in West Africa [27,28,29]. However, the roles, core competencies and minimum standards of CHWs are often not adequately addressed in local or national disaster risk management plans in health and other sectors [30]. When governments or organisations plan to establish core competency sets, it can be difficult to modify the generic ones used across different programmes and institutions for specific national use. Whilst there are sometimes competency frameworks in countries, these frameworks tend to focus on preparedness and response rather than holistic risk management aligned with the Health EDRM framework [22]. 

There is a need to ensure that global guidelines or frameworks evolve with developments in the field of Health EDRM. Therefore, it is important to define a range of evidence-based, practical, globally accepted core competencies and standardised knowledge frameworks that can be tailored to any country context to be integrated into national Health EDRM capacity development strategies, policies and plans according to population health priorities and any identified skills gaps [22,31,32].

Third, although there are wide-ranging Health EDRM workforce initiatives and programmes available globally, developing such programmes is a difficult task for many countries [24]. These difficulties may arise from many directions, including lack of understanding of the need, not being included in governmental priorities, limited physical and financial resources, lack of an established information exchange and coordination mechanisms (particularly evident in resource-poor countries), presence of post-conflict phases, people who live in remote locations, and communities with marginalised populations [33,34,35,36]. Moreover, even when programmes are available, they tend to focus on practical skills but often do not address management functions, for example, effective governance, coordination mechanisms, and human resource management among external support workers and the local workforce to maximise the use of the available health workforce [22,24]. Sharing best practices from existing Health EDRM workforce development strategies can illustrate how to plan, manage, retain, motivate, deploy, and coordinate human resources [31].

In response to the urgent needs to generate more evidence for policies and practices, the WHO called for proposals for the Health EDRM Workforce Development research project in 2019 [11]. This paper highlights the preparation and plan of this project, based on the proposal *‘Health workforce development strategy in health EDRM: evidence from literature review, case studies and expert consultations.’* This international project was initiated in June 2020. The aims and objectives of this comprehensive research project were developed in-line with the research questions identified by the WHO Thematic Platform for Health EDRM and its research group. This project aims to identify the recommendations and best practices for Health EDRM workforce development and to create recommendations facilitating effective Health EDRM workforce development strategies to inform policy and practice across WHO regions. Table 1 summarises research needs and rationales for this project.

## 4. Approach to Addressing the Evidence Gap

**Figure 1:**
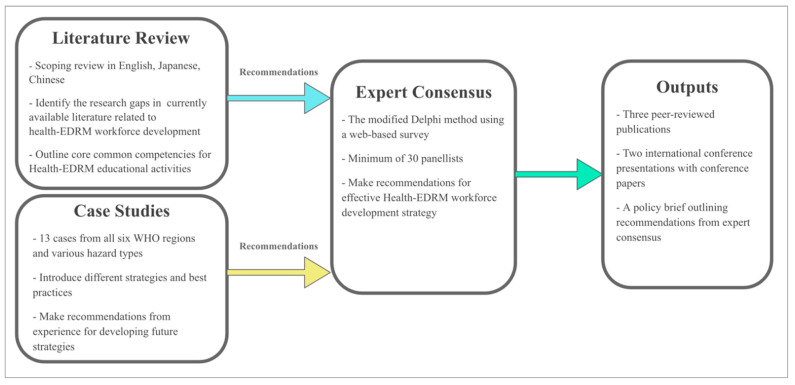
Flow chart of the study.

This project uses a multi-faceted research approach, involving literature reviews, case studies and an expert consensus study. Literature reviews and case studies will be completed simultaneously to generate policy and practice recommendations that will form the foundation for the consensus study’s questionnaires. This project’s flow chart is shown in Figure 1. Each study method is described in more detail below.

### 4.1. Participating Institutions

The project team is composed of experts from institutions in Hong Kong, China, Japan, the USA, Italy, the Philippines, Nepal, and India. All investigators have extensive knowledge and experience in disaster and emergency medicine, nursing, and public health. The roles and responsibilities of each institution were defined prior to the project as described in Table 2. Table 2 also shows the case studies that each institute will produce.

### 4.2. Literature Reviews

Existing literature related to health workforce development for Health EDRM in three languages (English, Chinese and Japanese) will be reviewed. A scoping review approach will be employed to synthesise and map search findings in a comprehensive and systematic way [37]. This approach was considered more suitable than a systematic review for this project, due to the heterogenous nature and large volume of existing literature on these topics [38]. The PRISMA extension (2018) for scoping reviews will be used for validation [39].

Literature searches use MEDLINE (1966), EMBASE (1980), CINAHL (1980) for English; ICHUSHI (1983) for Japanese; and CNKI (1976) for Chinese. Inclusion and exclusion criteria for the English literature review are listed in Table 3. Japanese and Chinese literature reviews will follow similar criteria. The initial title/abstract screening will be conducted by a single reviewer, then the full paper screening performed by two independent reviewers (with a third tie breaker if needed) to select final papers. Data will be extracted in standardised data extraction forms. Both quantitative data and qualitative output will be extracted.

The scoping exercise will identify relevant papers for this Health EDRM workforce development review. The findings should highlight research gaps and training needs for creating recommendations for developing Health EDRM workforce development strategies. The recommendations will also form the basis for the initial questionnaire to be given to the expert consensus study. 

### 4.3. Case Studies

Thirteen case studies will present various ongoing Health EDRM workforce development initiatives and programmes from low- and middle-income countries (LMIC) as well as high-income countries (HIC) in the six WHO regions. All initiatives were developed in relation to disasters occurring after 1995 and were included as suitable case studies drawing on the project team members’ experiences.

The combination of the selected case studies will illustrate wide-ranging Health EDRM workforce development approaches in different contexts. The cases will include primary and secondary data through literature reviews or key informant interviews as described in Table 4. Due to the differing nature of all cases, information sources will vary depending on data availability.

This multiple case study approach will include two separate analyses, ‘analysis within cases’ and ‘analysis across cases’ [40,41]. Each analysis will likely emphasise and convey different findings. 

First, a within-case analysis will be conducted for each case to provide in-depth information about the case, including programme description, facilitators/challenges, and recommendations for future programmes. Furthermore, each case will highlight important aspects of their initiatives, for example, coordination mechanisms or quality assurance systems. The in-depth analysis will provide insight into what worked, why and how. These case studies altogether will provide a holistic illustration of on-going Health EDRM workforce development activities and be good examples to share.

Second, an across-cases analysis will be performed to identify similarities and differences between each case. This will lead to empirical generalisability and theory development for Health EDRM-related workforce development. The findings from both types of analysis will contribute to the expert consensus study [42].

### 4.4. Expert Consensus Study

The final stage of this project will be an expert consensus study. The modified Delphi method, using an iterative web-based survey, will be conducted to seek consensus from a group of global Health EDRM experts to identify the strategic recommendations [43]. The modified Delphi methods enable a group of diverse experts to make decisions independently and anonymously without a face-to-face meeting [44]. It is a commonly used method to aggregate expertise opinions for health policy development or determining research priorities when available evidence is limited [33,45,46,47]. The first survey will be formed based on the results of the literature reviews and case studies. The final consensus on what strategies should be in the policy recommendations will be achieved after three rounds of surveys, analyses and feedback. Each questionnaire will be administered with one month in-between each round to allow selected panellists to familiarise themselves with the procedures. 

Expert panellists will be selected based on known expertise, prior publication records, relevant positions held in related institutions and on direct recommendations from other investigators in the field. The final panellists will include members both from within and outside of the project team and will comprise a minimum of 30 experts, 15 each for the LMIC and HIC groups. An equal gender balance will also be targeted.

Separate consensus plans and questionnaires will be developed for LMIC and HIC groups due to potential differences in their Health EDRM priorities and needs. The final strategic recommendations will be disseminated in a policy brief via the World Health Organisation (WHO) to inform national Ministries of Health.

### 4.5. Strengths and Limitations of the Current Methodology

We will conduct a scoping review in three languages: English, Japanese, and Chinese. The search terms in three languages are designed to be equivalent, however, they may be interpreted differently due to cultural and contextual differences. Therefore, we confirmed the terms among the literature review teams prior to beginning the reviews. We also standardised the selection criteria in Table 3 and used a similar data extraction form across all teams to ensure consistency.

Concerning the case studies, experiences from different types of disasters in LMIC and HICs are included. However, as each case study focuses on a single instance, generalisability and transferability of the findings will need to be carefully assessed before conducting across-case analyses and formulating strategic recommendations. Furthermore, to avoid inconsistencies, data collection and reporting guidelines are shared among the project team members. The Chinese University of Hong Kong team coordinates regular meetings to ensure open and timely information sharing and feedback among the investigators.

With travel restrictions in-place during the COVID-19 pandemic, a web-based voting system for the expert consensus was felt to be the best option. However, there may be issues involving panellists’ engagement due to a lack of background information for this project, which may hinder decision-making. Hence all selected panellists will be given a summary of this project, including the results of the literature reviews and case studies. We are also planning to present the literature reviews and the case studies at international conferences and recordings of the presentations will be made accessible to panellists. Furthermore, panellists will be carefully selected according to selection criteria to better define knowledge and experience.

## 5. Importance of Evidence-Based Recommendations for Health EDRM Workforce Development

The primary aim of this project is to produce a set of clear, concise and actionable recommendations for facilitating effective Health EDRM workforce development strategies. It is anticipated that the recommendations will be useful supports for diverse audiences in guiding future Health EDRM workforce development at local, national and regional/global level. The potential impacts at different level of society are summarised in Table 5.

### 5.1. Local/Community Level

Hazardous events of all scales, including disasters, can happen in both rural and urban communities and directly threaten the health of communities. Disaster risk is projected to rise due to hazard modifiers (e.g., climate change) and growing exposures and vulnerabilities (e.g., unplanned urbanisation) [3]. Hence, in addition to a well-established national-level approach to disaster risk reduction, building capacity to cope with the risks of emergencies with strong community participation is also essential to reducing both the risk and impact of disasters. The Health EDRM framework emphasises a people- and community-centred approach as people in communities are often the first responders in emergencies and their local knowledge and experience are imperative for the successful implementation of Health EDRM initiatives [10]. The One Billion Coalition for Resilience [48] and the 2015 World Disasters Report [49] by the International Federation of Red Cross and Red Crescent Societies (IFRC) also highlighted the importance of empowering local communities for resilience-building. 

Community members should be central in community-based ‘bottom-up’ or ‘grassroots’ initiatives [50]. From the outset, it is recommended to engage local stakeholders who can be involved in risk assessment and programme implementation [51]. They can help collect data on specific local disaster risks as well as local knowledge, culture, and health risks. Crucially, they can also promote programme participation and implementation in their communities [52]. In order for communities to prevent, prepare for and respond efficiently to the sudden onset of disasters, community residents require adequate knowledge and skills through training or risk communication. 

A ‘bottom-up’ planning approach begins with working with communities to address the actual needs and priorities in the target community. This is done by identifying local hazards, vulnerabilities and capacities, leading to the development of specific activities to mitigate identified local risks and increasing local capacities to address risks that affect communities, including sub-populations who are at higher risks [51,53]. Various community-based health promotion activities have been developed following this pathway, for example dealing with non-communicable diseases and mental health [52,54,55,56]. Nevertheless, the roles and support of national and regional actors remain important for community disaster risk management because inputs from governmental bodies are essential for providing adequate regulations, financial resources and technical capacities [57].

Enhancing community disaster resilience requires coordinated efforts among all key stakeholders, including community leaders who could identify priorities and train the local workforce according to local needs. Multidisciplinary partnerships are an important coordination mechanism for successful ‘bottom-up’ initiatives as they can yield greater impacts by combining the expertise of every project stakeholder [52,58,59]. Support from governments and partners is also crucial to reinforce the community health workforce by strengthening existing health systems and providing resources (such as funding, technical support, human resources, or supplies) [30].

Making the maximum use of capacities and capabilities of existing community health workers (CHWs) will also be a strong asset for community disaster risk assessment, preparedness, response, recovery, and sustainable resilience. CHWs have detailed knowledge about health needs and underlying vulnerabilities in their communities and hold high levels of local trust which enhance their credibility and legitimacy [60]. Hence, strengthening community-based Health EDRM actions that emphasise community engagement is very important.

The case studies and recommendations developed in this project can be used as evidence and guidance to assess the available resources and capacities in communities for developing or revising community-based disaster preparedness and response plans. The recommendations from this project may encourage national governments to formalise a ‘people- and community-centred approach’ in their national health workforce development plans aligned with the Health EDRM Framework. This project will provide tools to develop, implement and manage community Health EDRM workforce development strategies, including setting core competencies and minimum standards for community workforce training programmes [61].

### 5.2. National Level

Effective national health systems and health service coverage depend on the availability, accessibility, acceptability, and quality of health workers, especially during emergencies when health needs of the population are likely to surge [62,63]. The COVID-19 pandemic highlighted that responding to acute and chronic health needs from disasters and emergencies could result in significant infrastructure and service disruption even in HICs with well-established health systems. Therefore, in order for a country to establish or scale up health workforce quality and availability during emergencies, there is a strong need for national governments to develop or revise the national health and multisectoral DRM plans and health workforce strategies. This process requires comprehensive strategic approaches with careful planning. 

Our project will include a health workforce capacity assessment tool to determine national priorities, such as the available health workforce, underserved areas, required skills, available training and supervision, as well as any imbalances between workforce and health needs anticipated during health emergencies. This set of priorities will help identify the most urgent and suitable solutions to implement for a country. A strong multisectoral partnership is also crucial to maximise institutional capacity, including sufficient funding, appropriate policy, and legal frameworks, well-established coordination mechanisms, and clear accountability. The series of case studies will illuminate the best practices needed to accommodate functional collaboration aimed at strengthening a national Health EDRM workforce.

### 5.3. Regional/Global Level

Some regional and international organisations provide training programmes targeting specific professionals or create frameworks to define roles and responsibilities of professionals. 

The WHO Regional Offices have conducted regional Health EDRM workforce training through various programmes, e.g., PHEMAP (Public Health and Emergency Management in Asia and the Pacific), PHEMEURO (Public Health and Emergency Management in Europe); and MPHR (Management of Public Health Risks in Disasters and Complex Emergencies) which have largely been discontinued. These programs responded to the challenge that the majority of available training programmes tended to focus on technical practices, on specific diseases, or on preparedness and response, but not comprehensive all hazards risk management. Therefore, training is still required to further address management functions for emergencies and disasters globally and as well using the global standards at national and regional levels.

As an example at the global level, the WHO Emergency Medical Team (EMT) initiative aims to promote minimum standards for surge capacity. Since 2015, a global registry for EMTs has been implemented to help governments ensure that only qualified teams are deployed in affected areas. EMTs must undertake a quality assurance process, showing they have well trained staff and appropriate protocols, equipment and supplies to provide quality care adapted to the context [24,64]. 

The International Council of Nurses (ICN) and the WHO developed a Framework of Disaster Nursing Competencies to enhance nursing workforce capacities during global health emergencies. The ICN/WHO framework serves as a common set of competencies for the global disaster nursing workforce and provides clarification of nurses’ roles in disasters at local, national, and international levels [65,66]. Both the EMT and the Disaster Nursing Competencies will be fully illustrated in our case studies.

As highlighted in the ongoing pandemic of COVID-19, international initiatives such as OpenWHO and Global Outbreak Alert and Response Network (GOARN) serve an important role as a global disaster risk communication and information sharing platform to observe and manage large scale epidemics [67,68]. OpenWHO was developed by WHO in 2020 and is a web-based interactive knowledge-transfer platform, offering timely and evidence-based scientific information and learning during health emergencies. The OpenWHO.org platform has grown significantly with more than 5 million learner registrations. The number of courses and learners has grown significantly with 25 free online COVID-19 courses in 44 languages. 

GOARN developed a global risk communication and information management platform in 2020 called the ‘COVID-19 knowledge hub’ to provide updated information on COVID-19 to the Health EDRM workforce to enable more effective disaster risk management, including training activities and infection prevention and control measures [69]. The COVID-19 pandemic demonstrates the essential role of Health EDRM workforce development. Providing evidence and best practices serves to facilitate and strengthen the network of international stakeholders, and foster effective coordination, alignment and accountability to tackle the global challenges in Health EDRM workforce development.

## 6. Conclusions

This comprehensive research programme, consisting of literature reviews, case studies, and an expert consensus study, aims to provide strategic recommendations for health workforce development strategies in Health EDRM. Key common competencies and knowledge required in training or education curricula will emerge from the literature reviews. Case studies will present existing Health EDRM workforce initiatives in various contexts. The strength of this project lies in the detailed planning and comprehensive approach to this important but diverse topic. The final strategic recommendations will inform national Ministries of Health and should provide pathways to review national workforce capacities and plan for building a stronger global Health EDRM workforce.

## Figures and Tables

**Table 1:** Research Needs and Rationales.

Research Needs	Rationales
1. A need to define Health EDRM workforce and to characterise the profile and scope of groups that constitute Health EDRM workforce	- No generally accepted definition - Hard to find a clear division of roles between groups
2. A need to categorically define evidence-based, globally accepted core competencies and standardised knowledge frameworks	- Lack of global guidelines or frameworks with a set of standard competencies for developing training programmes - Programmes often use varying competencies, terminologies and course structures - Some programmes may not be developed based on evidence - The inconsistent standards between programme could cause fragmented humanitarian assistance to affected communities - The roles, core competencies and minimum standards of community health workers often not addressed in local or national DRM plans - It can be difficult to modify the generic ones used across different programmes and institutions for specific national use - Competency frameworks in countries tend to focus on preparedness and response rather than holistic risk management
3. A need to share experience from existing Health EDRM workforce development strategies	- Challenges in developing Health EDRM workforce initiatives and programmes for many countries - Programmes tend to focus on practical skills but often do not address management functions: for example, coordination mechanisms, retention, deployment

**Table 2:** The Roles and Case Studies of Participating Universities.

University	Literature Review	Case Studies	Expert Consensus
The Chinese University of Hong Kong, Hong Kong SAR, China	Overall coordination and delivery of the project Leading the English and Chinese literature review	Overall coordination of case studies and the creation of 3 case studies: 1. Pharmacy workforce in post-conflict sub-Saharan African countries 2. Community health workers for future disasters in Nepal 3. Community disaster education initiatives in rural China	Overall coordination of the Delphi research
Harvard University, USA		4. Health workforce demands in Lebanon 5. Earthquake response teams in Chile	All participants will contribute their expertise in study design and be invited as a panellist as appropriate
Sichuan University, China		6. Logistics Support of Emergency Medical Teams	
Tohoku University, Japan	Supporting the Japanese literature review	7. Multidisciplinary conductor type disaster health workforce development program 8. Competency framework of Japan DMAT and specialised assistance teams	
University of Hyogo, Japan	Leading the Japanese literature review	9. Disaster relief nursing in Japan	
University of Piemonte Orientale, Italy		10. Undergraduate medical training in disaster medicine 11. Emergency Medical Teams (EMTs) Training	
University of the Philippines Manila, Philippines		12. Disaster nursing training and management in the Philippines	
WHO India, India		13. Hospital preparedness and planning in India	
Outputs	Literature review in 3 languages 1 peer reviewed publication 1 international conference presentation	13 case studies 1 peer reviewed publication 1 international conference presentation	Expert consensus recommendations Policy brief

**Table 3:** Inclusion and Exclusion Criteria.

Inclusion Criteria	Written in English (Japanese or Chinese) and published from 1990 to 11 Mar 2020 Addressing the health risks associated with emergencies and disasters (e.g., attributable to biological, natural, technological, societal hazards, human-induced disasters including acts of mass violence and terrorism) Including findings concerning health workforce development initiatives of Health EDRM
Exclusion Criteria	Health workforce development activities based primarily on military setting Studies describing the training of one single type of clinical procedure or surgery Focusing mainly on the experience/processes conducting research in disaster settings Conference abstracts, letter or editorial without full reporting of data Full text not available Not written in English (Japanese or Chinese)

**Table 4:** Case studies and their data source.

Cases	Description	Data Source
1: Pharmacy workforce in post-conflict sub-Saharan African countries	Make recommendations to better inform pharmacy workforce development policies in post-conflict areas	Literature review Key informant interviews
2: Community health workers for future disasters in Nepal	Explore the roles of female community health volunteers during and following the 2015 earthquake	Literature review Key informant interviews
3: Community disaster education initiative in rural China	Describe planning and implementation process of a Health EDRM education initiative in China	Literature review Personal experience of programme managers/implementers
4: Health workforce demands in Lebanon	Describe how Lebanon’s health system and workforce coped with a rapid 25% population increase	Literature review Key informant interviews
5: Earthquake response teams in Chile	Describe the training regimens and best practices from the experience of the Earthquake Response in Chile	Literature review Discussion with responders
6. Logistic Support for Emergency Medical Teams in China	Summarise the experience, lessons and development of logistics support	Literature/policy reviews
7. Multidisciplinary conductor type disaster health workforce development program	Review the comprehensive disaster training programme in Japan, focusing on its development and deployment	Literature review Personal Experience
8. Competency framework of Japan DMAT and specialised assistance teams.	Identify good practice and gaps in the education programme	Literature review
9. Disaster Relief Nursing in Japan	Describe disaster relief nurse programme in Japan focusing on training, registration, dispatch and operation	Literature review Personal experience
10. Undergraduate medical training in Disaster Medicine	Present a disaster medicine training programme and discuss its cost-effective and reproducible solutions	Literature review Personal experience
11. Emergency Medical Teams (EMTs) Training	Highlight coordination and quality assurance mechanisms for the training programme	Literature review Personal experience
12: Disaster nursing training and management in the Philippines	Describe a national training of trainers’ programme in disaster nursing management in the Philippines	Literature review Key informant interviews
13. Hospital Emergency Preparedness and Planning in India	Summarise safe hospital initiatives in India	Literature/policy reviews Personal experience

**Table 5:** Potential Impacts from this project at different levels of society.

Level	Potential Impacts of Recommendations
Local/Community	1. Enhancing community disaster resilience and capacity development by promoting a people and community centred approach
	2. Encouraging the development of community-based Health EDRM initiatives
	3. Making the maximum use of capacities and capabilities of community health workers
	4. Providing evidence to develop or revise community-level disaster risk management plans
National	5. Providing evidence or guidance to develop or revise the national DRM and health workforce plans and strategies
	6. Helping to determine national priorities and the most suitable solutions to implement for countries
	7. Illustrating the best practices to accommodate effective multisectoral partnership for strengthening a national Health EDRM workforce
Regional/Global	8. Providing evidence and best practices to facilitate and strengthen the network of international stakeholders
	9. Fostering effective coordination, alignment and accountability to tackle the global challenges in Health EDRM workforce development
